# Differences in Intestinal Microbiota Between White and Common Cranes in the Yellow River Delta During Winter

**DOI:** 10.3390/biology14060704

**Published:** 2025-06-16

**Authors:** Xiaodong Gao, Yunpeng Liu, Zhicheng Yao, Yuelong Chen, Lei Li, Shuai Shang

**Affiliations:** 1 College of Life Sciences, Qufu Normal University, Qufu 273165, China; gao-xiaodong@163.com; 2 College of Biological and Pharmaceutical Engineering, Shandong University of Aeronautics, Binzhou 256600, China; hmlyp@163.com; 3 Zibo Forestry Protection and Development Center, Zibo 255000, China; yaozhicheng1985@163.com; 4 Nanjing Hongshan Forest Zoo, Nanjing 210018, China; yuelong1988chen@163.com

**Keywords:** intestinal microorganisms, *Grus*, LEfse, 16S rDNA

## Abstract

The avian intestinal microbiota is a vital interface for host/environment interactions, playing a pivotal role in nutrient metabolism, immune regulation, and ecological adaptation. In the Yellow River Delta region, common cranes and white cranes coexist in mixed flocks. During the winter, when food resources are scarce, studying their gut microbiota can effectively reveal the feeding patterns of these two crane species, thereby providing valuable data for crane conservation efforts. This study systematically investigated and compared the intestinal microbiota structures of white cranes (*Grus leucogeranus*) and common cranes (*Grus grus*) inhabiting the Yellow River Delta region. The results demonstrated that the predominant phyla of the intestinal microbiota in white and common cranes are Firmicutes and Proteobacteria at the phylum level; *Catellicoccus* and *Lactobacillus* were the predominant genera in the crane species. LEfse was used to analyze the differential flora of the intestinal bacterial communities of white cranes and common cranes and to detect the marker species with significant differences between the groups. Based on the COG database, a preliminary functional prediction of the intestinal microbiota was conducted, and 16 metabolic pathways relating to the COG pathway were obtained. In general, although both types of cranes belong to the *Grus* genus and are distributed in the same area, there are significant differences in the composition and functional characteristics of their intestinal microbiota due to the differences in their feeding composition.

## Simple Summary

This study investigated the differences in intestinal microbiota composition between white cranes and common cranes in the Yellow River Delta region. The results demonstrate that the predominant phyla of the intestinal microbiota in both white and common cranes are Firmicutes and Proteobacteria, indicating that these phyla play a crucial role in maintaining the normal physiological functions of the *Grus* species. At the genus level, *Catellicoccus* was identified as the predominant genus in the *Grus* species. Notably, *Catellicoccus* is also dominant in the gut microbiome of murres and is commonly found across various avian species. However, significant disparities exist in the composition of the gut microbiota between the two crane species. We hypothesize that despite both species belonging to the genus *Grus* and inhabiting the same region, differences in their feeding habits likely contribute to notable variations in the composition and functional characteristics of their intestinal microbiota.

## 1. Introduction

Animal intestines provide an optimal living environment for various microorganisms. The intestinal microbiota forms a complex, integrated system comprising diverse microbial communities [1,2]. Intestinal microorganisms and the host collaboratively establish a relatively stable internal environment characterized by symbiosis and mutual benefit [3]. Studies have demonstrated that maintaining a balanced gut microbiota enhances animal immunity and ensures overall health [4]. In particular, the avian intestinal microbiota is a vital interface for host/environment interactions, playing a pivotal role in nutrient metabolism, immune regulation, and ecological adaptation [5]. In recent years, the widespread adoption of high-throughput sequencing technology has facilitated the rapid advancement of research on avian gut microbiota [6]. Evidence indicates that the composition of the avian intestinal microbiota is closely associated with genetic background, diet, migratory behavior, and habitat environment [7,8]. Notably, the microbial dynamics of migratory birds are particularly significant due to their frequent traversal of geographical barriers and diverse habitat types [7]. For instance, *Grus* spp.—a flagship species of wetland ecosystems—exhibits intestinal microbial diversity and functional characteristics that not only serve as biological indicators of host health but also reflect environmental stressors in their habitats and the stability of the ecosystem [9,10]. However, current studies predominantly focus on single species or captive populations, leaving comparative analyses of sympatric species under natural conditions relatively underexplored.

Today, the impact and damage caused by global environmental problems on natural ecosystems are becoming increasingly severe, which in turn affects the intestinal microbiota of birds [11,12]. This not only affects the survival of birds but also relates to the success or failure of reintroduction [13]. Therefore, attending to the diversity of the gut microbiota in wild birds holds substantial research and ecological significance. Investigating the impact of host and environmental factors on the gut microbiota of wild birds and elucidating the relationship between their gut microbiota and ecological adaptation can facilitate a more profound comprehension of the physiological mechanisms underlying the co-evolution of gut microorganisms and their hosts, which can provide a robust theoretical foundation for preserving biodiversity and conserving ecological environments. The white crane (*Grus leucogeranus*) and the common crane (*Grus grus*), keystone species along the East Asia–Australasia Flyway, exhibit exceptionally high conservation significance. The white crane is classified as Critically Endangered (CR) by the International Union for Conservation of Nature (IUCN), with a global population estimated at fewer than 4000 individuals. Its survival is critically contingent upon preserving key wetland ecosystems, such as the Yellow River Delta. While the common crane has a broader distribution range, its traditional habitats are increasingly threatened by agricultural expansion and wetland fragmentation. Notably, dietary differentiation between the two species is pronounced: white cranes predominantly consume aquatic plants and benthic invertebrates, whereas common cranes primarily forage on leftover grains in farmlands during the wintering season [10,14]. This dietary divergence may influence the functional differentiation of gut microbiota via trophic interactions. Current research indicates that the gut microbiota of cranes is predominantly composed of Firmicutes, Proteobacteria, and Actinobacteria; however, the composition of core genera and the underlying environmental response mechanisms across species remain poorly understood [14,15,16].

As the most intact wetland ecosystem in the global warm temperate zone, the Yellow River Delta serves as a critical “international hub” for migratory birds and a natural laboratory for investigating the interactions between environments, hosts, and microorganisms [17]. In recent years, restoration initiatives, such as farmland-to-wetland conversion and ecological water replenishment, have expanded the wetland area within the protected zone by 188 square kilometers, increasing the number of bird species from 187 to 410. The distinctive environment of the Yellow River Delta, where saltwater and freshwater converge, has fostered a unique vegetation pattern that indirectly influences the structure of avian gut microbiota through food resource availability and the physicochemical properties of water and soil. Although prior research has elucidated the compositional characteristics of the intestinal bacterial communities in cranes, several critical scientific questions remain unanswered: (1) How does the high-salt wetland environment of the Yellow River Delta influence the functional adaptability of the intestinal microbiota in white cranes and common cranes? (2) Does dietary variation and differential habitat utilization result in distinct patterns of pathogen carriage and resistance gene expression between these two species? This study focuses on the white and common crane within the Yellow River Delta Nature Reserve. By employing non-invasive fecal sampling coupled with 16S rRNA high-throughput sequencing and metabolic function prediction, we systematically analyze the compositional differences in the intestinal bacterial communities of the two species and their underlying driving mechanisms. This investigation reveals the co-adaptive strategies of crane intestinal microbiota in the unique habitat of the Yellow River Delta. The findings not only enhance our understanding of the co-evolutionary mechanisms between birds and microorganisms but also supply foundational data on microbial diversity for the ecological management of the Yellow River Estuary National Park, thereby supporting the conservation of endangered species and the sustainable use of wetlands.

## 2. Materials and Methods

### 2.1. Study Area and Sample Collection

The Yellow River Delta region is characterized by its abundant wetland resources and a strategically unique geographical location, situated at the convergence of the coastal plain and coastal tidal flats. Surrounded by extensive farmland, this area serves as a critical habitat for numerous wildlife species and functions as an important wintering ground for cranes (38.0617° N, 118.6669° E). In the Yellow River Delta region, the foraging behaviors of common cranes and white cranes were systematically observed at predetermined sites. Notably, populations of red-crowned and common cranes have shown significant recovery. Nevertheless, the rapidly changing wetland environment and anthropogenic activities (e.g., *Spartina alterniflora* invasion and agricultural non-point source pollution) may threaten the stability of crane gut microbiota by altering food chain structures and elevating the risk of pathogen transmission. In January 2025, observations were conducted from approximately 500 m away using 10 × 60 binoculars, while relevant image data were recorded using cameras. For the sampling process, we divided ourselves into two teams: one focused on monitoring the activities of white cranes, while the other monitored gray cranes. Primary emphasis was placed on observing their foraging behavior and defecation patterns. Researchers promptly proceeded to the foraging area after the crane flocks had completed their foraging activities and departed. Wearing disposable gloves, they used tweezers to collect fresh fecal samples within a straight-line distance of approximately 5 m from the ground. Approximately 6–8 g of each sample was collected from the upper portion. Following collection, the samples were placed in sterile centrifuge tubes, labeled with detailed information, and accompanied by records of the sampling environment parameters. GPS devices were utilized for precise positioning and documentation. In the present study, 20 samples were obtained from two groups. Ten common crane samples were named BH1-10, and ten white crane samples were named DYB1-10. The collected samples were temporarily stored in a portable refrigeration unit and transferred to a laboratory refrigerator on the same day for preservation. Finally, after sampling, the samples were transferred to a −20 °C refrigerator for long-term storage in preparation for DNA extraction.

### 2.2. DNA Sample Extraction and Sequencing Analysis

The fecal samples identified as being from gray cranes were all sent to the sequencing company, where DNA was extracted from each fecal sample of common cranes and white cranes. The 16S rDNA gene region was amplified by PCR (95 °C for 2 min, followed by 30 cycles at 95 °C for 30 s, 55 °C for 30 s, 72 °C for 30 s, and a final extension at 72 °C for 5 min) using primers 515F 5′-GTGCCAGCMGCCGCGG-3′ and 907R 5′-CCGTCAATTCMTTTRAGTTT-3′ [18]. PCR products were extracted and purified before being quantified by Qubit^®^3.0. After the company extracted the total DNA samples of feces, the samples were also subjected to electrophoresis detection using a microplate spectrophotometer and 1.20% agarose gel. Based on the concentration of the sample after testing, DNA samples of unity were diluted to 20 ng/mol, according to the grouping of DNA sequencing project samples, and mixed with the corresponding primers for PCR amplification. The fragment size and concentration were quantified after library preparation, and high-throughput sequencing was carried out on an Illumina NovaSeq 6000 platform (Biomarker Technologies Co., Ltd., Beijing, China).

### 2.3. Data Analysis

The paired-end sequencing of the corresponding amplification products was conducted using the Illumina MiSeq platform. The sequencing data were stored in FASTQ format. Based on project requirements, two processing methods were applied to the data—the DADA2 method and an alternative approach. For the DADA2 method, the QIIME2 software was initially employed to remove the primers and unmatched sequences [19]. Subsequently, quality control, denoising, stitching, and dechimerization were performed by invoking the DADA2 program [20]. High-quality sequences were clustered and denoised, partitioned into amplicon sequence variants (ASVs) (hereinafter collectively referred to as features), and the taxonomic classification of each feature was determined based on its sequence composition. Based on the feature analysis results, a comprehensive taxonomic analysis was performed on the samples across multiple classification levels. This analysis yielded community structure diagrams, species clustering heatmaps, phylogenetic trees, and taxonomic tree diagrams for each sample at the phylum, class, order, family, genus, and species levels. Using QIIME2 software, a rarefaction curve was generated by randomly subsampling sequences from each sample to provide a rough estimate of species diversity across samples. Statistical analysis of species composition at the species classification level was performed based on sequence classification annotations for different analysis items. Functional composition diversity prediction was carried out using PICRUSt2 software [21]. During group analysis, the relative abundance content represented the average of all relative abundance values for grouped samples. Alpha diversity analysis was used to reflect species diversity within a specific geographical area. Several metrics are commonly used to quantify alpha diversity, including Chao1, Shannon, Simpson, and Coverage. The Chao1 and Ace indices specifically estimate species richness, which refers to the total number of distinct species present in the sample. In contrast, the Shannon and Simpson indices measure species diversity, taking into account both species richness and the evenness of their distribution within the community. Principal component analysis (PCA) was used to analyze and simplify complex datasets by decomposing variance and representing the differences among multiple sets of data on a two-dimensional coordinate graph. Linear Discriminant Analysis Effect Size (LEfSe) is a method capable of simultaneously conducting differential analysis across all taxonomic levels to identify robust biomarkers distinguishing groups. LEfSe analysis was applied to investigate differences between groups of intestinal bacteria and their relationships. Diversity analyses were based on results processed using the Python LEfse package (Version 2.7).

## 3. Results

### 3.1. Bacterial Community Diversity

**Figure 1:**
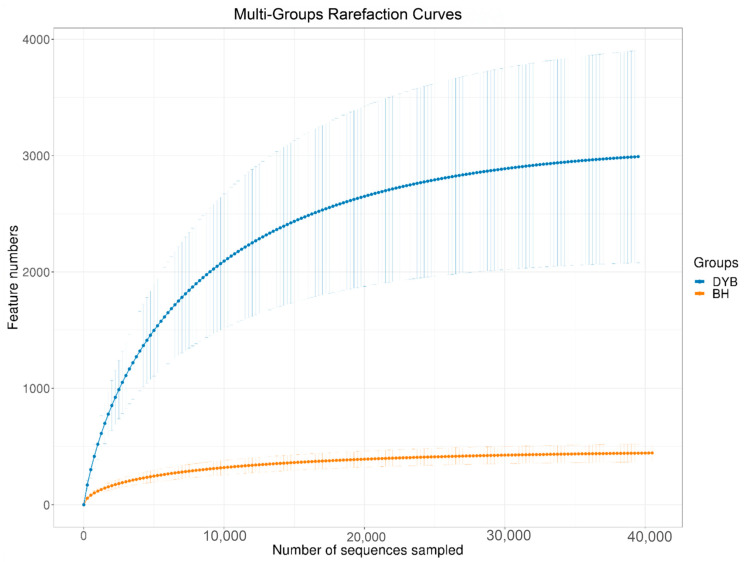
Multi-group rarefaction curves between two groups. DYB: white crane group; BH: common crane group.

This study’s 20 samples were sequenced to obtain 1,391,493 paired-end reads. Following quality control and assembly, 1,235,801 clean reads were generated and classified into 29,563 amplicon sequence variants (ASVs). A total of 618,296 clean reads were obtained in the common crane, and 617,505 clean reads were obtained in the white crane. The observed rarefaction curve and Shannon diversity index curve, presented in Figure 1, indicate that the sequencing depth was sufficient, the data coverage exceeded 99%, and the sample sequences adequately represented the microbial community structure. These results confirm the sequencing data’s reliability in reflecting the samples’ accurate composition.

**Figure 2:**
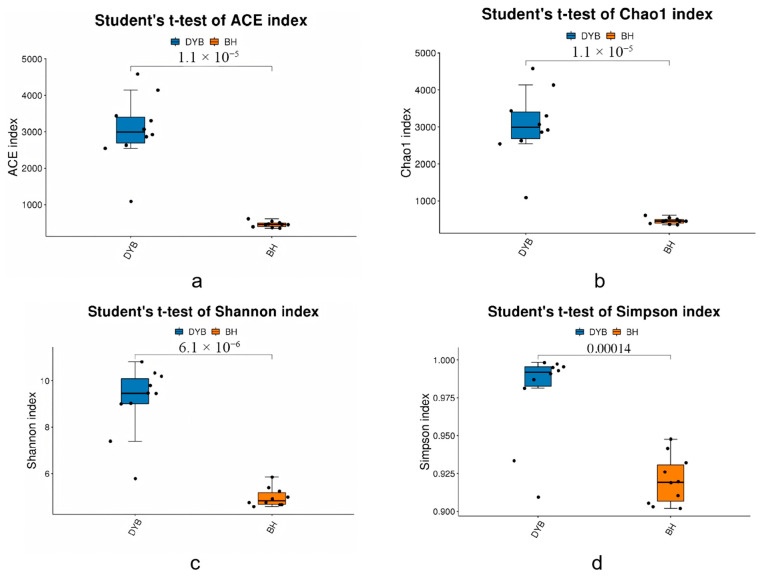
α-diversity analysis index of two groups. (**a**) ACE index; (**b**) Chao1 index; (**c**) Shannon index; (**d**) Simpson index; DYB: white crane group (10 samples); BH: common crane group (10 samples).

The α-diversity analysis of bacterial communities across the 20 samples is summarized in Figure 2 and based on indices that include ACE, Chao1, Simpson, and Shannon. The Ace (Figure 2a) and Chao1 (Figure 2b) indices are employed to estimate species richness, defined as the total number of distinct species within a community. The Shannon (Figure 2c) and Simpson (Figure 2d) indices are used as quantitative measures of species diversity, which is influenced by the richness of both species and the evenness of species distribution within the sample community. At a given level of species richness, an increased evenness among species corresponds to a higher perceived diversity. Higher values of the Shannon and Simpson indices reflect greater levels of species diversity within the sample. Significant differences were detected between the white crane and the common crane groups (*p* < 0.01, Student’s *t*-test), suggesting that the bacterial communities associated with white cranes exhibited a higher degree of richness and diversity than those of common cranes.

**Figure 3:**
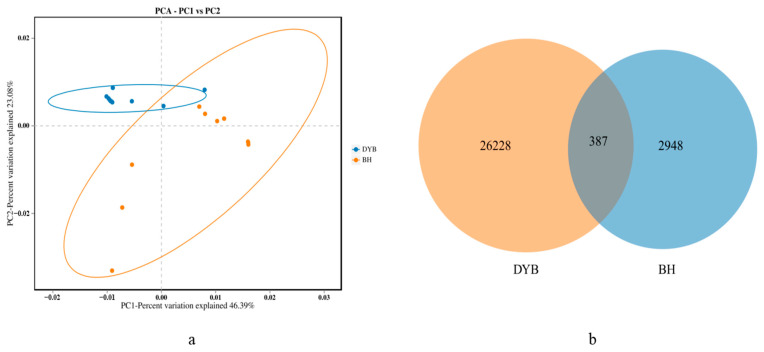
Venn diagram analysis among groups (**a**) and PCA (**b**). (**a**): Structural differences in bacterial communities among the groups were assessed using PCA based on the weighted UniFrac distance metric. The value in percentage indicates the contribution of PC1 to the variability. Y-axis: Second principal component. The value in percentage indicates the contribution of PC2 in variability; (**b**): DYB: white crane group; BH: common crane group.

PCA based on unweighted UniFrac distance was performed to determine the difference in the group composition structure. PCA1 and PCA2 explained 23.08% and 46.39% of the variations, respectively (Figure 3a). The PCA results showed clear separations between the two groups, representing the difference in bacterial communities. In addition, samples from the same group were clustered together, while samples in the white crane group showed a higher degree of similarity compared to those in the common crane group. A Venn diagram was used to illustrate the number of shared and unique amplicon sequence variants (ASVs) between the white cranes and common cranes. By integrating the species represented by these ASVs, it was possible to identify common microorganisms across different environments. The results (Figure 3b) demonstrated that the common crane had 2948 unique ASVs, while the white crane had 26,228 unique ASVs, with 387 ASVs being shared between the two species. Our results suggest that the bacterial community associated with white cranes comprises more ASVs and exhibits a higher degree of species diversity.

### 3.2. Bacterial Community Composition

**Figure 4:**
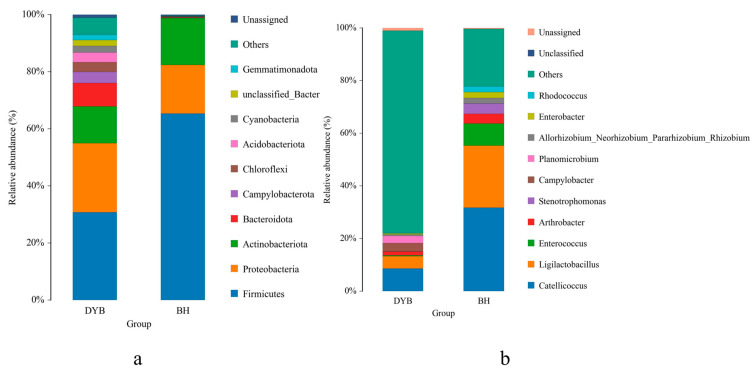
Relative abundance of the dominant bacterial at the phylum (**a**) and genus (**b**) levels. Note: X-axis: sample IDs; Y-axis: relative abundance. DYB: white crane group; BH: common crane group. Unassigned means the organism/sequence was analyzed but cannot be placed into any defined taxonomic group; unclassified means the organism/sequence lacks sufficient data for taxonomic placement, often due to technical limitations; Others: a catch-all category for sequences that do not fit into predefined categories of interest in the study.

The relative abundance of the bacterial community at the phylum level is shown in Figure 4a. The dominant groups in the relative abundance of bacterial communities in the intestinal tract of the white crane are Firmicutes (30.77%), Proteobacteria (24.16%), Acidobacteriota (12.91%), and Bacteroidota (8.16%). These four phyla constitute 76% of the intestinal microbiota in white cranes. The dominant groups in the relative abundance of bacterial communities in common cranes are Firmicutes (65.39%), Proteobacteria (16.98%), and Acidobacteriota (16.41%). The cumulative percentage of the three bacterial phyla reached 98.78% in common cranes.

In terms of the relative abundance at the genus level, as shown in Figure 4b, the white crane’s intestinal composition favors *Catellicoccus* (8.61%), *Ligilactobacillus* (4.68%), *Campylobacter* (3.22%), and *Planomicrobium* (2.68%). The dominant genera among the common cranes are *Catellicoccus* (31.74%), *Ligilactobacillus* (23.55%), *Enterococcus* (8.36%), *Arthrobacter* (3.69%), and *Stenotrophomonas* (3.94%). The *Catellicoccus* and *Ligilactobacillus* genera were dominant in both groups.

### 3.3. LEfse Analysis

**Figure 5:**
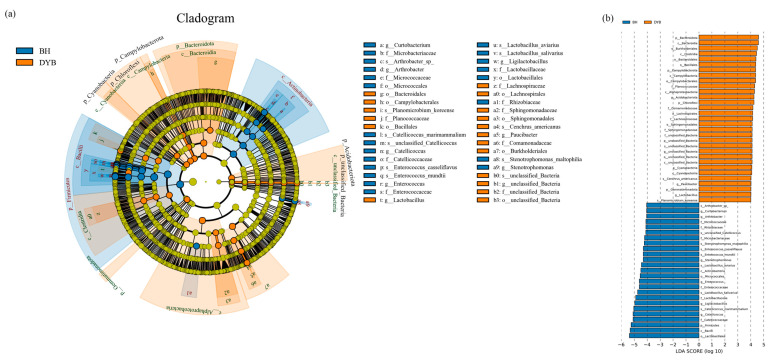
LEfSe analysis of the specific bacterial community between the two groups. (**a**) LEfSe biomarkers in cladogram. (**b**) LEfSe biomarkers in strict. Note: the circles from the center to the outward layers represent taxonomic levels from phylum to species. The node on the circles represents a term on the corresponding taxonomic level. The size of the dots indicates relative abundance. Coloring: Species with no significant difference are colored in yellow. Otherwise, the nodes are colored according to the group with the highest relative abundance, which helps visualize the relevance of different biological aspects. DYB: white crane group; BH: common crane group.

LEfse was used to analyze the differential flora of white cranes and common cranes’ intestinal bacterial communities and detect the marker species with significant differences between the groups. The results are shown in Figure 5. At the phylum level, Firmicutes were significantly enriched in the intestinal tract of common cranes, while Bacteroidota, Campylobacterota, Acidobacteriota, Chloroflexi, and Cyanobacteria were significantly enriched in white cranes. At the genus level, *Curtobacterium*, *Arthrobacter*, *Stenotrophomonas*, *Enterococcus*, *Ligilactobacillus*, and *Catellicoccus* were significantly enriched in the intestines of common cranes. In white cranes, *Paucibacter* and *Lactobacillus* were significantly enriched.

### 3.4. Function Prediction

**Figure 6:**
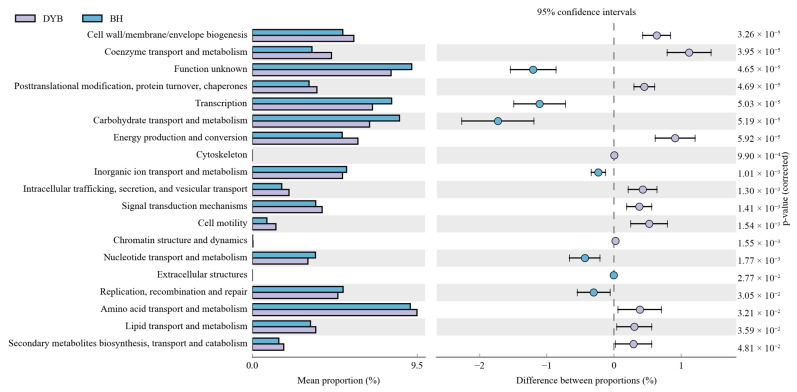
PICRUSt2 predicts microbial metabolic functions through COG analysis between the two groups. Note: the left figure shows the abundance proportion of different functions in two groups of samples, the middle figure shows the difference in proportion of functional abundance within the 95% confidence interval, and the value on the far right is the *p*-value. DYB: white crane group; BH: common crane group.

Using PICRUSt2 (Phylogenetic Investigation of Communities by Reconstruction of Unobserved States), we analyzed the functional differences in bacterial communities in white cranes and common cranes based on the Clusters of Orthologous Groups (COGs) database (Figure 6). Sixteen significant functional differences were identified between white and common cranes in the intestinal bacterial communities. Specifically, functions, such as cell wall/membrane/envelope biogenesis, coenzyme transport and metabolism, post-translational modification, protein turnover, chaperones, energy production and conversion, intracellular trafficking, secretion, vesicular transport, signal transduction mechanisms, cell motility, amino acid transport and metabolism, lipid transport and metabolism, secondary metabolites biosynthesis, and transport and catabolism, were more prominent in the intestinal microbiota of common cranes. In contrast, functions, including transcription, carbohydrate transport and metabolism, ion transport and metabolism, nucleotide transport and metabolism, replication, recombination, and repair, were more significant in the intestinal microbiota of white cranes.

## 4. Discussion

Birds represent a highly successful group of organisms characterized by rich species and genetic diversity [22]. In contrast to mammals, the gut microbiota of birds exhibits lower stability and greater plasticity [23]. Specifically, the relatively short rectum in birds results in undigested food being directly excreted as feces without storage. This rapid digestive process renders the avian gut microbiota more susceptible to external influences and prone to adjustment [24]. Furthermore, bird nests, which display significant variability and harbor complex microbial communities [25], play a crucial role in shaping the gut microbiota of chicks during early development [26]. Additionally, wild birds exhibit diverse dietary preferences, flight behaviors, and developmental strategies [27], further contributing to the complexity of their gut microbiota. Research has demonstrated that alterations in the gut microbiota of wild birds not only influence host physiological traits, nutritional status, and stress responses [24] but also enhance ecological adaptability through dynamic regulatory mechanisms [28].

**Figure 7:**
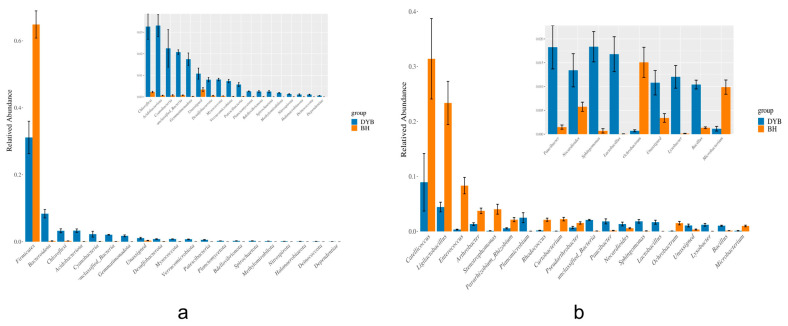
Statistical analysis of differences in gut microbiota composition at the phylum and genus levels. DYB: white crane group; BH: common crane group. (**a**) illustrates the analysis of differences in bacterial phyla between the two groups at the phylum level; (**b**) depicts the analysis of differences between the two groups at the genus level.

The results indicate that the predominant phyla of the intestinal microbiota in white and common cranes are Firmicutes and Proteobacteria, suggesting that these phyla play a critical role in sustaining the normal physiological activities of *Grus* species. Existing research has demonstrated that the phylum Firmicutes is ubiquitously present in the gut microbiota and assists the host in breaking down complex carbohydrates, polysaccharides, and fats [29], thereby enhancing energy metabolism. Actinobacteria, which are commonly found in soil, water, and air [30], are also prevalent in the gut microbiota of numerous bird species [31]. Additionally, the phylum Proteobacteria exhibits diverse physiological functions and plays a pivotal role in the host’s utilization of carbon sources and energy storage, contributing to meeting the elevated energy and nutritional demands of organisms [32]. Compared with the common crane, Bacteroidota, Campylobacterota, Chloroflexi, and Acidobacteriota were most dominant in the white crane intestines (Figure 7). Bacteroidota comprise one of the most dominant microbiota in the animal intestine, maintaining the host’s energy balance by regulating sugar and fat metabolism, affecting the development of the immune system, and inhibiting inflammatory responses [33]. Chloroflexi, also known as green-bending bacteria, fulfill a range of significant roles in the human body and the environment. Within the human body, Chloroflexi predominantly reside in the digestive tract, performing functions such as degrading polysaccharides to facilitate digestion, producing metabolic byproducts, and contributing to the nitrogen cycle [34]. Their involvement in the nitrogen cycle is critical for maintaining nitrogen equilibrium in the digestive tract and the broader ecosystem. We posit that the intestinal microbiota of the white crane sample group could break down a greater amount of nutrients in food, thereby enhancing the efficiency of food utilization, providing increased energy for the host organism, and supporting the host’s survival. At the genus level, *Catellicoccus* was the predominant genus of crane species and was dominant in the murre gut microbiome and ubiquitous in the gut microbiomes of various avian species [35]. *Catellicoccus* encodes various functions, such as nutrient transport and bile acid hydrolysis, suggesting a symbiotic lifestyle for this species [35]. Possible beneficial effects for the host, such as immune modulation and gut maturation, have also been proposed for bacteria of this genus inhabiting the avian gut [36]. Meanwhile, *Lactobacillus* was dominant in two species, which aligns with findings from studies on crane species, such as the white-headed crane [37] and the demoiselle crane (*Anthropoides virgo*) [38]. *Lactobacillus*, a member of the phylum Firmicutes, is a probiotic in the animal gut [39] and is highly abundant in the host’s gastrointestinal tract. It aids digestion and absorption, maintains intestinal flora homeostasis, reduces serum cholesterol levels, and enhances immune function [40]. A previous study demonstrated that supplementing *Lactobacillus* can mitigate intestinal flora imbalances induced by *Escherichia* coli infections in ducks [41]. In this study, the abundance of *Ligilactobacillus* was significantly higher in the common crane compared to the white crane sample group. We posit that common cranes’ digestive efficiency, nutrient absorption, and immune capabilities might surpass those of white cranes.

Previous studies have found that the intestinal microbiota adapts to dietary changes in birds during the wintering period. Before the wintering period, the white-headed crane (*Grus monacha*) and the black-necked crane mainly feed on rice, and the *Prevotella* genus, which is involved in the decomposition and metabolism of plant proteins, is the dominant bacteria in their intestines, participating in multiple metabolic functions [37,40]. Based on the COG database, a preliminary functional prediction of the intestinal microbiota was conducted, and 16 metabolic pathways of the COG pathway were obtained. During the overwintering period of common cranes, crops are their main component. The intestinal bacteria of common cranes mainly comprise Firmicutes and Proteobacteria, which may be related to their intake of high carbohydrates (such as grains) [14]. During the overwintering period of the white crane, wetland plants and mollusks are the main species. Besides Firmicutes and Proteobacteria, Bacteroidota, Campylobacota, Chloroflexi, and Acidobacteriota also account for significant proportions. In the inter-group difference analysis of the top five metabolic pathways in relative abundance, it was found that cell wall/membrane/envelope biogenesis, coenzyme transport and metabolism, post-translational modification of the white crane sample group protein turnover, chaperones, energy production and conversion, intracellular trafficking, secretion, and vesicular transport, signal transduction mechanisms, cell motility, amino acid transport, and metabolism, the relative abundances of lipid transport and metabolism, secondary metabolites biosynthesis, and transport and catabolism were all higher than those of the gray crane sample group. The intestinal flora of the white crane sample group can decompose more nutrients—such as proteins in food—enhance the utilization rate of food, provide more energy for the body, and help the host maintain survival. These results indicate that the gut microbiota can regulate metabolic pathways to adapt to different host environments. The transcription, carbohydrate transport and metabolism, ion transport and metabolism of common cranes, the relative abundances of nucleotide, transport and metabolism, and replication, recombination, and repair were all higher than those of the white crane sample group, which is related to the fact that the common crane mainly feeds on herbivorous food.

## 5. Conclusions

This study investigated the differences in intestinal microbiota composition between white cranes and common cranes in the Yellow River Delta region. The results demonstrate that the predominant phyla of the intestinal microbiota in both white and common cranes are Firmicutes and Proteobacteria, indicating that these phyla play a crucial role in maintaining the normal physiological functions of *Grus* species. At the genus level, Catellicoccus was identified as the predominant genus in the *Grus* species. Notably, *Catellicoccus* is also dominant in the gut microbiome of murres and is commonly found across various avian species. However, significant disparities exist in the composition of the gut microbiota between the two crane species. We hypothesize that despite both species belonging to the genus *Grus* and inhabiting the same region, differences in their feeding habits likely contribute to notable variations in the composition and functional characteristics of their intestinal microbiota.

## Data Availability

All sequences analyzed in the present study can be accessed in the SRA database under the accession number SUB15335174, BioProject lD: PRJNA1265934.
